# Spectrum-Efficacy Relationships between GC-MS Fingerprints of Essential Oil from Valerianae Jatamansi Rhizoma et Radix and the Efficacy of Inhibiting Microglial Activation

**DOI:** 10.1155/2022/9972902

**Published:** 2022-03-07

**Authors:** Nan-Yun Yang, Qi-Rui Li, Xu Zhang, Fan-Li Zeng, Xiang-Chun Shen, Qing-De Long

**Affiliations:** ^1^The State Key Laboratory of Functions and Applications of Medicinal Plants (Department of Medicinal Plants and Pharmacognosy), School of Pharmaceutical Science, Guizhou Medical University, Huaxi University Town, Guian New District 550025, Guizhou, China; ^2^The Key Laboratory of Utilization of Natural Medicines (The High Efficacy Application of Natural Medicinal Resources Engineering Center of Guizhou Province, The High Educational Key Laboratory of Guizhou Province for Natural Medicinal Pharmacology and Druggability), School of Pharmaceutical Science, Guizhou Medical University, Huaxi University Town, Guian New District 550025, Guizhou, China

## Abstract

The bioactive ingredients of essential oil from Valerianae Jatamansi Rhizoma et Radix (the Rhizome et Radix from Valerianae Jatamansi Jones) (EOVJRR) on the efficacy of inhibiting microglial activation were investigated with the approach of spectrum-efficacy relationship. Fourteen batches of Valerianae Jatamansi Rhizoma et Radix were extracted and analyzed by gas chromatography-mass spectrometry (GC-MS), and their activities in the efficacy of inhibiting microglial activation were assayed by measuring the inflammatory responses induced by lipopolysaccharide (LPS) in microglia cells from mice. The spectrum-efficacy relationships between fingerprints and the efficacy of inhibiting microglial activation of EOVJRR were established by grey relational analysis (GRA). Twenty common peaks were obtained from the GC-MS fingerprints of EOVJRR. P12 (vetivenol), P1 (bornyl acetate), P5 (seychellene), and P3 (*β*-elemene) indicated inhibition on microglia activation together, according to the spectrum-efficacy relationships. The current results established a general model for the spectrum-efficacy relationships of EOVJRR by GC-MS and the efficacy of inhibiting microglial activation, which could be applied to identify the bioactive ingredient and control the quality of herbs.

## 1. Introduction

Inflammation of the central nervous system (CNS) is well known to play a crucial role in depression and other neurodegenerative diseases [1–3]. Sleeplessness, anhedonia, despair, and helplessness are typical symptoms of depression, which is becoming increasingly prevalent (common) throughout the world as society develops [4]. To date, the pathogenesis mechanism of depression is not understood in detail, and many pathophysiological factors contribute to the development of depression. Among these factors, inflammatory factors are highlighted in the progress of depression [5, 6]. Some antidepressants have been widely applied in clinics to alleviate depressive symptoms; however, these drugs exhibit therapeutic benefits and bring serious adverse effects [7]. Therefore, it is urgent to clarify the pathophysiology of depression and develop novel antidepressant drugs.

Microglial cells are the innate immune cells in the nervous system. Growing evidence has shown that microglial activation is involved in the pathogenesis of neurodegenerative and psychiatric disorders [8–10]. The overactivation of microglial was accompanied by body areas growing larger and protruding pseudopods, as well as increasing the secretion of proinflammatory factors, such as interleukin-1 beta (IL-1*β*) [11] and interleukin-6 (IL-6) [12]. Microglial activation has been reported to be associated with depression [13–15]. Lipopolysaccharide (LPS) is an endotoxin derived from the outer membranes of Gram-negative bacteria, which can stimulate microglia to switch to an active state and activate the inflammatory pathway [16]. Peripheral injection of LPS could induce microglial activation in the brain and induce a depressive state, accompanied by increased expression of inflammatory factors. Microglia had been identified as a key therapeutic target in major depressive disorder [17, 18].

Valerianae Jatamansi Jones is belonging to the Valerianaceae family [19], which is known as “Zhizhuxiang” in China. It is a tiny, perennial dwarf, hairy, rhizomatous herb having thick roots covered with fibers and distributes from Afghanistan to southwest China, India, Nepal, Bhutan, and Myanmar at an altitude of 1000 to 3000 m [20–22]. Valerianae Jatamansi Rhizoma et Radix is the Rhizome et Radix from Valerianae Jatamansi Jones, which is indexed in Chinese Pharmacopeia (Part 1) in 1977, 2010, 2015, and 2020 editions as a traditional Chinese medicine [23]. It is commonly used in the intervention and treatment of mental diseases such as depression [24], anxiety, epilepsy, and insomnia [25]. V. Jatamansi Rhizoma et Radix has a special aroma, which is derived from the essential oil extracted from it. EOVJRR has been shown to have (possess) anti-inflammatory and antioxidant activity in previous investigations [26, 27]. Therefore, we investigated the effect of EOVJRR on LPS-stimulated microglia in vitro and explored the active ingredients.

The spectrum-efficacy relationship is regarded as a systematic approach to determine the effective components of traditional Chinese medicines (TCMs) [28]. There are varieties of analytical methods performed to establish a spectrum-efficacy relationship, including grey relational analysis (GRA), partial least squares regression (PLSR), and principal component analysis (PCA) [29, 30]. Among them, GRA is a beneficial method for indistinct system research, which contains both known information and unknown information. It has the characteristics of a small sample size, low requirements of data distribution rule, and easy programming. It can determine the size of the relevance between the efficacy index and the chromatographic peak, as well as provide a possibility for predicting the effective components [31]. Simultaneously, it can overcome the limitations of statistical analysis methods such as regression and correlation that require large amounts of data and clearly define data distribution features. Therefore, it has been extensively used in the study of the spectrum-efficacy relationship and quality control evaluation of TCMs with complex chemical ingredients. To further elucidate the active ingredients in EOVJRR that inhibit the effect of microglial activation, we now established the GC-MS fingerprint and compared the efficacy of inhibiting microglial activation of different batches of EOVJRR through pharmacological studies of microglial cells. The correlation between the common peaks and the efficacy of inhibiting microglial activation was analyzed by GRA. According to the grey relational grade, the effective components were clarified and the contribution of each ingredient to the efficacy of inhibiting microglial activation was determined, which indicated that the EOVJRR is a potential candidate for inflammation-induced depressive symptoms.

## 2. Materials and Methods

### 2.1. Materials

The fourteen batches of fresh Rhizome et Radix from V. Jatamansi Jones were obtained from Guizhou Province in October 2019, and their species were identified by Professor Qingde Long (School of Pharmaceutical Sciences, Guizhou Medical University). Their origins were listed as follows: Guiyang city (S1), Weining city (S2), Duyun city (S3), Panzhou city (S4), Liuzhi city (S5), Hezhang city (S6), Anshun city (S7), Tongren city (S8), Zunyi city (S9), Meitan city (S10), Chishui city (S11), Xingyi city (S12), Kaili city (S13), and Jinsha city (S14).

### 2.2. Reagents

Microglial cells were purchased from Shanghai Mingjin Biotechnology Co., Ltd. (Shanghai, China). Fetal bovine serum and DMEM/F-12 medium were purchased from Gibco in the USA. Lipopolysaccharide (LPS) and 3-(4,5-dimethylthiazol-2yl)-2,5-diphenyl tetrazolium bromide (MTT) were purchased from Sigma-Aldrich (St. Louis, MO, USA). TransZol Up Plus RNA Kit, EasyScript® One-Step gDNA Removal and cDNA Synthesis SuperMix and TransStart® Top Green qPCR SuperMix were purchased from Transgen (Beijing, China). IL-6 and IL-1*β* primer were purchased from Shanghai Shenggong Biological Engineering Co., Ltd. (Shanghai, China).

### 2.3. EOVJRR Preparation

The essential oil from V. Jatamansi Rhizoma et Radix (EOVJRR) was extracted by steam distillation. The dry Rhizome et Radix from V. Jatamansi Jones was crushed. The crushed powder (200 g) was placed into the round-bottomed flask with 2000 mL water and subjected to steam distillation extraction. The progress of extraction was taken 7 h [32–34], 14 batches of EOVJRR were sealed and stored at −20°C after treating with anhydrous sodium sulfate (Na_2_SO_4_).

### 2.4. Analysis of GC-MS Fingerprints

The GC-MS analysis of EOVJRR was performed with fused silica capillary column samples that were injected into a 7890A/5975C-GC/MSD system (Agilent, USA) with ChemStation workstation and NIST mass spectrometry database. The chromatographic separation was performed using a DB-5MS gas chromatography column (30 m × 250 *μ*m × 0.25 *μ*m, Agilent, USA). The temperature of the oven program was firstly raised to 40°C and maintained for 5 min, and then temperature was increased from 40°C to 280°C at a rate of 5°C/min and that temperature was maintained for 5 min. Helium was used as carrier gas at a constant flow rate of 1.0 mL/min. EI mode at 70 eV was used as the ionization mode of the spectrometers with a source temperature of 230°C. The split ratio was 40 : 1; the mass spectra plot was obtained with a scan range from 20 to 450 m/z. The components were identified by comparing their mass spectra with data from NIST (National Institute of Standards and Technology, US). The sample injection volume is 1.0 *μ*L.

### 2.5. Inhibiting Microglia Activation Determination

#### 2.5.1. Drug Preparation and Groups

EOVJRR was diluted into a concentration of 10 mg/mL with DMSO, filtered and sterilized with a 0.22 *μ*m microporous membrane, stored at −20°C in the refrigerator, and used medium to dilute progressively (DMSO concentration is less than 0.0001%).

Microglial cells were cultured in DMEM/F-12 medium solution containing 10% fetal bovine serum and placed in an incubator at 37°C saturated humidity and 5% CO_2_. The cultured cells were designed in different groups as follows: control group, LPS-reduced model (1 *μ*g/mL) group, and 14 batches EOVJRR groups. Preincubated with the EOVJRR, LPS was exposed and incubated for 6 h for establishing an acute microglial activation model.

#### 2.5.2. MTT Assay

Microglial cells were seeded in a 96-well plate and cultured for 24 h and treated with 14 batches of EOVJRR and 1 *μ*g/mL LPS; the experiment was repeated 3 times with 3 wells of each group. After being exposed to LPS for 6 h, MTT was added to each well and placed in an incubator for 4 h; the supernatant was discarded and dissolved by DMSO. Finally, the 96-well plate was used to determine the optical density (OD) of each well at 490 nm. The cell viability of the EOVJRR to microglial cell was calculated by the following formula:

(1) $$\begin{array}{c} \mathrm{cell}\;\mathrm{viability}\left(\%\right)=\frac{\left(\mathrm{OD}\;\mathrm{of}\;\mathrm{experimental}\;\mathrm{group}\;\right)}{\left(\mathrm{OD}\;\mathrm{of}\;\mathrm{negative}\;\mathrm{control}\right)}\times 100\%. \end{array}$$

#### 2.5.3. ELISA Assay

The ELISA assay method was used to evaluate the efficiency of inhibiting microglial activation of EOVJRR against the LPS-induced microglial activation model. Microglial cells were incubated in a 96-well plate and cultured for 24 h to detect the secretion of IL-6 and IL-1*β*. The group and administrative methods were identical to those used in 1.5.1. To remove particles and polymers, collect the cell supernatant of each group and centrifuge at 3000 r/min for 10 min. Set up standard wells and sample wells, and fill each standard well with 50 *μ*L of different concentration standards. Firstly, add 10 *μ*L of the sample to be tested, and 40 *μ*L of sample diluent to the sample wells, then add 100 *μ*L of HRP-labeled detection antibody to each well, and seal the reaction well and incubate in a 37°C incubator for 1 h. Next, discard the liquid, pat dry on absorbent paper, fill each well with washing solution for 1 min, then pat dry on absorbent paper again, repeat this for 5 times, and then add 50 *μ*L of substrate A and B to each well to incubate at 37°C in the dark for 15 min. Finally, add 50 *μ*L of stop solution to each well, and measure the OD value of each well at 450 nm within 15 min.

#### 2.5.4. Quantitative Real-Time PCR

Quantitative real-time PCR (qRTPCR) was used to evaluate the efficacy of inhibiting microglial activation of EOVJRR. Microglial cells were incubated in a 6-well plate and cultured for 24 h. The group and administrative methods were identical to those used in 1.5.1. Extract total RNA by TRIzol method, determine the concentration and purity of RNA, and then synthesize cDNAs by reverse transcription. cDNAs were amplified using the following conditions: hold 94°C/30 s, denaturation 94°C/5 s, annealing 60°C/15 s, and final extension 72°C/10 s, as directed by the manufacturer. The qRTPCR method was used to determine the mRNA levels of IL-6 and IL-1*β*, with GAPDH serving as an internal control to normalize the amount of RNA in each sample, and the sequence of the primers is shown in Supplementary Table S1. The calculations of the relative amount of transcripts were performed using the ΔCT method [35].

### 2.6. Spectrum-Efficacy Relationship

Spectrum data were analyzed using GC-MS, and the inhibitory effect of EOVJRR in the microglial was evaluated by ELISA and qRTPCR to detect the secretion and mRNA levels of IL-6 and IL-1*β*. Grey relational analysis was used to establish the spectrum-efficacy relationship between GC-MS fingerprints and the efficacy of inhibiting microglial activation.

#### 2.6.1. Data Preprocessing

In GRA [36], initial data preprocessing is performed to transform the original data sequences, with different measurement units, into comparable sequences. The secretion and mRNA levels of IL-6 and IL-1*β* were considered as a reference sequence, and the common characteristic peak areas of EOVJRR were used as a comparison sequence. Standardize the data with Z-score before performing grey relational analysis. The reference sequences and comparison sequences are represented by {*X*_0_ (*k*)} and {*X*_*i*_ (*k*)}, *i* = 1, 2,…, *m*; *k* = 1, 2,…, *n,* respectively, where *m* is the number of experiments and *n* is the total number of observations of data.

#### 2.6.2. Grey Relational Grade Calculation

The grey relational coefficient was calculated from the deviation sequence using the following relation:

(2) $$\begin{array}{c} \gamma \left(x_{0}\left(k\right),x_{i}\left(k\right)\right)=\frac{\min_{i}\min_{k}\left|x_{0}\left(k\right)-x_{i}\left(k\right)\right|+\xi \max_{i}\max_{k}\left|x_{0}\left(k\right)-x_{i}\left(k\right)\right|\;}{\left|x_{0}\left(k\right)-x_{i}\left(k\right)\right|+\xi \max_{i}\max_{k}\;\left|x_{0}\left(k\right)-x_{i}\left(k\right)\right|},\;0<\gamma \left(x_{0}\left(k\right),x_{i}\left(k\right)\right)\ll 1. \end{array}$$

*ξ* is the resolution coefficient, usually *ξ* ∈  (0,1). The resolution coefficient is typically chosen to be 0.5. A grey relational grade is the weighted average of the grey relational coefficient and is defined as follows:

(3) $$\begin{array}{c} \gamma \left(X_{0},\;X_{i}\right)=\;\frac{1}{n}\sum_{k=1}^{n}\gamma \left(x_{0}\left(k\right),x_{i}\left(k\right)\right). \end{array}$$

The grey relational grade between the reference sequence and comparison sequences was calculated. The grey relational grade is higher, the correlation between the chemical composition and the efficacy is greater [37].

### 2.7. Statistical Analysis

All the data were analyzed using GraphPad Prism 6.01 software (GraphPad Software, CA) and presented as the mean ± SEM. One-way analysis of variance (ANOVA) was carried out for multiple comparisons of the data, and *p* < 0.05 indicated that the difference was statistically significant.

## 3. Results and Discussion

### 3.1. GC-MS Analysis of EOVJRR

**Figure 1:**
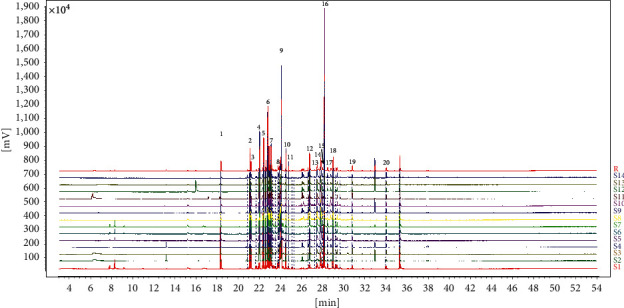
The GC-MS fingerprints of 14 batches of essential oil from V. Jatamansi Rhizoma et Radix (EOVJRR) and 20 common peaks. The chromatograms of S1–S14 represent as follows: Guiyang city, Guizhou (S1), Weining city, Guizhou (S2), Duyun city, Guizhou (S3), Panzhou city, Guizhou (S4), Liuzhi city, Guizhou (S5), Hezhang city, Guizhou (S6), Anshun city, Guizhou (S7), Tongren city, Guizhou (S8), Zunyi city, Guizhou (S9), Meitan city, Guizhou (S10), Chishui city, Guizhou (S11), Xingyi city, Guizhou (S12), Kaili city, Guizhou (S13), Jinsha city, Guizhou (S14), and reference standard fingerprint (R), respectively.

The chemical fingerprints of EOVJRR were achieved by GC-MS, and the results are shown in Figure 1. Twenty common peaks of GC-MS were determined using Similarity Evaluation System for Chromatographic Fingerprint of TCM (Version 2012). The identification of EOVJRR was achieved through a library search in an MS library (NIST17).

The identified results of 20 common compounds are shown in Table 1, and it was listed that the coefficients of variance (C.V.%) for 20 common characteristic peaks were at the range of 20.76–68.76%. It implied that the content of each composition in 14 batches of samples differed significantly, as did the intrinsic quality of the samples.

### 3.2. Similarity of Fingerprints

The similarity between the fingerprint of 14 batches of EOVJRR and the reference fingerprints respectively was at the range of 0.621–0.985 (Table 2), which closely matched expected S2. The similarity between S2 and the other samples was all less than 0.827, which indicated that there was a large difference between S2 and the other samples. Therefore, it can be concluded that the production area had a significant impact on the content and chemical components of EOVJRR.

### 3.3. Results of Principal Component Analysis

**Figure 2:**
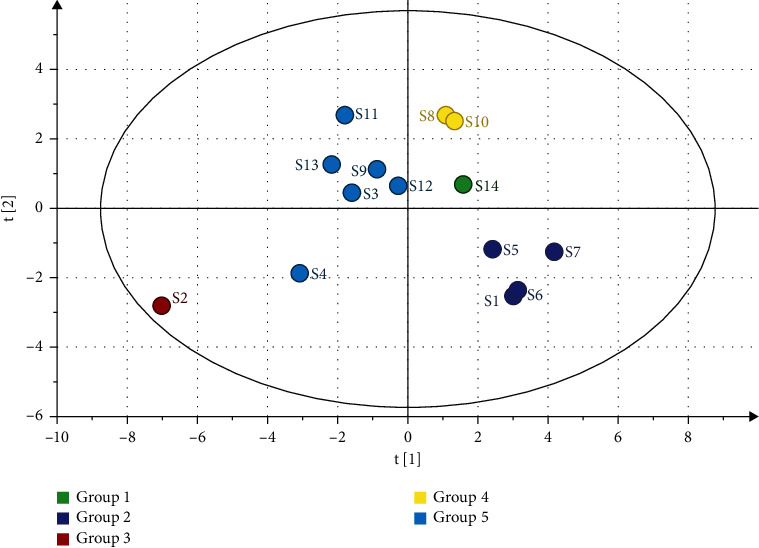
Principal component analysis of 14 batches of EOVJRR from various sources.

As illustrated in Figure 2, 14 batches of EOVJRR samples could be grouped into five categories, S14 belongs to group one, S1, S5, S6, and S7 belong to group two, S2 belongs to group three, S8 and S10 belong to group four, S3, S4, S9, S11, S12, and S13 belong to group five. Therefore, it could conclude that some critical factors, such as area and growth environment, could play the same important role in influencing the quality of V. Jatamansi Jones.

### 3.4. Effect of EOVJRR in Microglial Cells

**Figure 3:**
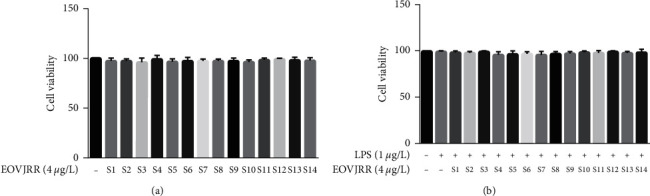
Cell viabilities of 14 batches of EOVJRR (a) and LPS (b) in microglial cells ($\bar{x}$ ± *s*, *n* = 3).

MTT was used to detect the toxicity of EOVJRR in microglial cells. As shown in Figure 3, the cell viability of each group was more than 95% after being treated with LPS (1 *μ*g/mL) and 14 batches of EOVJRR (4 *μ*g/L). This suggested that LPS (1 *μ*g/mL) and EOVJRR (4 *μ*g/L) had no toxicity in the microglial cells.

IL-6 and IL-1*β* were involved in microglia activation. As shown in Table 3, the efficacy of inhibiting microglial activation of 14 batches of EOVJRR was tested by the secretion and mRNA expression of IL-6 and IL-1*β*. The secretion and mRNA levels of IL-6 and IL-1*β* were found to be significantly higher in the vehicle group than in the normal group (*p* < 0.01 and *p* < 0.001). Different batches of EOVJRR could suppress the secretion and mRNA levels of IL-6 and IL-1*β*. It can be seen that different areas resulted in inhibiting microglia activation. The analysis results were as follows: (1) with secretion of IL-6 used as an index, there was no obvious difference between S5, S8, S9, and vehicle group (*p* > 0.05), while the others had a greater inhibition effect with significant difference (*p* < 0.05 and *p* < 0.01); (2) with secretion of IL-1*β* used as an index, all of EOVJRR from various sources had greater inhibition effect with significant difference compared with the vehicle group (*p* < 0.05 and *p* < 0.01); (3) with IL-6 mRNA level used as an index, between S2, S5, S8, S9 and vehicle group had no obvious difference (*p* > 0.05), while the others had a greater effect with significant difference (*p* < 0.05 and *p* < 0.01); (4) with IL-1*β* mRNA used as an index, S2 had no obvious difference (*p* > 0.05) that compared with the vehicle group, and the others had greater inhibition effect with significant difference compared with the vehicle group (*p* < 0.05 and *p* < 0.01). The results of the combined efficacy of the different batches of EOVJRR showed that the effects of EOVJRR from Hezhang city (S6), Chishui city (S11), and Xingyi city (S12) were the optimal collection origin.

### 3.5. The Results of the Spectrum-Efficacy Relationships

GRA can examine the correlation of each peak and the efficacy of inhibiting microglial activation directly. As shown in Table 4, the correlation from high to low was P12 > P1 > P5 > P3 > P19 > P9 > P18 > P11 > P4 > P6 > P16 > P10 > P20 > P17 > P7 > P13 > P8 > P14 > P15 > P2. All common components made a significant contribution in inhibiting microglia activation with grey relational grade more than 0.9 expected P2 and P15. There was a certain relationship between the chemical components in the GC-MS fingerprint and the given component's inhibiting microglia activation. These results also indicated the efficacy of inhibiting microglial activation derived from the cooperative action of EOVJRR multiple components.

In summary, the efficacy of inhibiting microglial activation of EOVJRR was mainly affected by the synergistic effect with bornyl acetate, *β*-elemene, *α*-guaiene, seychellene, humulene, *α*-patchoulene, *β*-selinene, *α*-bulnesene, *α*-panasinsene, kessane, vetivenol, isovalencenol, *β*-humulene, patchouli alcohol, longifolen aldehyde, aristol-1(10)-en-9-yl isovalerate, (E)-valerenyl isovalerate, and neocembrene. Among them, P12 (vetivenol), P1 (bornyl acetate), P5 (seychellene), and P3 (*β*-elemene) were found to have a significant impact on the efficacy of inhibiting microglial activation of EOVJRR. The previous study reported that seychellene could inhibit the production of IL-6, IL-1*β,* and TNF-*α* [38]. *β*-Elemene could suppress the inflammatory progress through the Wnt/*β*-catenin signaling pathway [39] as well as protect the neuronal cells from injury through the anti-inflammatory pathway [40]. Bornyl acetate could regulate inflammatory cytokines [41] and treat atherosclerosis by mitigating the expression of proinflammatory cytokines [42].

The pathogenesis of depression is complex and diverse; stimulating the inflammatory pathway on microglial cells is one of the important mechanisms in patients with depression. In our work, we found that EOVJRR could inhibit the expression of inflammatory cytokines and inhibit microglial activation. Based on the spectrum-efficacy relationship, we discovered bioactive components of inhibiting microglial activation, such as vetivenol, bornyl acetate, seychellene, and *β*-elemene. This is the first time active components in essential oil from V. Jatamansi Rhizoma et Radix have been screened for inhibiting microglia activation.

## 4. Conclusion

In this study, the spectrum-efficacy relationship between pharmacodynamics of inhibiting microglial activation and GC-MS fingerprint has been successfully established to evaluate the internal quality of V. Jatamansi Jones and to explore its bioactive compounds. The results indicated that 20 common peaks were identified from 14 batches of EOVJRR. The major components that were primarily responsible for the efficacy of inhibiting microglial activation of EOVJRR were discovered as vetivenol, bornyl acetate, seychellene, and *β*-elemene. The further research should be focused on the pharmacodynamics and mechanisms of these active components. This is the first time, to our knowledge, that the spectrum-efficacy relationship of EOVJRR has been supported by screening active components of inhibiting microglial activation with GC-MS fingerprints of EOVJRR and providing a theoretical basis for future research.

## Figures and Tables

**Table 1:** The 20 common peak areas of 14 batches EOVJRR from various sources.

Peak number	Retention time (min)	Compounds	Molecular formula	CAS	C.V. (%)
1	18.334	Bornyl acetate	C_10_H_20_O_2_	2445-77-4	67.05
2	21.068	*β*-Patchoulene	C_15_H_24_	514-51-2	50.99
3	21.173	*β*-Elemene	C_15_H_24_	515-13-9	68.76
4	22.361	*α*-Guaiene	C_15_H_24_	3691-12-1	48.24
5	22.769	Seychellene	C_15_H_24_	20085-93-2	32.59
6	22.902	Humulene	C_15_H_24_	6753-98-6	31.83
7	23.065	*α*-Patchoulene	C_15_H_24_	560-32-7	28.68
8	23.747	*β*-Selinene	C_15_H_24_	17066-67-0	30.05
9	24.057	*α*-Bulnesene	C_15_H_24_	3691-11-0	38.59
10	24.494	*α*-Panasinsene	C_15_H_24_	56633-28-4	27.93
11	24.74	Kessane	C_15_H_26_O	3321-66-2	53.64
12	26.705	Vetivenol	C_15_H_24_O	68129-81-7	50.31
13	27.416	Isovalencenol	C_15_H_24_O	22387-74-2	29.15
14	27.778	*β*-Humulene	C_15_H_24_	116-04-1	35.11
15	27.955	*γ*-Gurjunene	C_15_H_24_	22567-17-5	40.67
16	28.121	Patchouli alcohol	C_15_H_26_O	5986-55-0	67.3
17	28.432	Longifolen aldehyde	C_15_H_24_O	66537-42-6	34.16
18	28.941	Aristol-1(10)-en-9-yl isovalerate	C_20_H_32_O_2_	—	44.88
19	30.755	(E)-Valerenyl isovalerate	C_20_H_32_O_2_	—	44.98
20	33.98	Neocembrene	C_20_H_32_	31570-39-5	20.76

C.V. (%) = *σ*/*μ* ∗ 100, *σ* is the standard deviation and *μ* is the average value of peak area.

**Table 2:** Similarities among EOVJRR from 14 various sources.

	S1	S2	S3	S4	S5	S6	S7	S8	S9	S10	S11	S12	S13	S14	*R*
S1	1.000	0.427	0.839	0.748	0.989	0.993	0.997	0.961	0.890	0.937	0.939	0.931	0.866	0.851	0.943
S2	0.427	1.000	0.751	0.827	0.495	0.485	0.473	0.410	0.705	0.375	0.585	0.625	0.710	0.673	0.621
S3	0.839	0.751	1.000	0.954	0.898	0.886	0.864	0.780	0.976	0.715	0.912	0.922	0.965	0.949	0.950
S4	0.748	0.827	0.954	1.000	0.820	0.806	0.780	0.694	0.941	0.621	0.836	0.873	0.936	0.953	0.903
S5	0.989	0.495	0.898	0.820	1.000	0.997	0.994	0.947	0.936	0.907	0.956	0.955	0.915	0.908	0.974
S6	0.993	0.485	0.886	0.806	0.997	1.000	0.996	0.943	0.923	0.906	0.947	0.947	0.904	0.898	0.964
S7	0.997	0.473	0.864	0.780	0.994	0.996	1.000	0.963	0.911	0.936	0.950	0.947	0.889	0.875	0.957
S8	0.961	0.410	0.780	0.694	0.947	0.943	0.963	1.000	0.851	0.988	0.937	0.920	0.842	0.808	0.913
S9	0.890	0.705	0.976	0.941	0.936	0.923	0.911	0.851	1.000	0.800	0.947	0.972	0.982	0.965	0.985
S10	0.937	0.375	0.715	0.621	0.907	0.906	0.936	0.988	0.800	1.000	0.905	0.884	0.783	0.733	0.867
S11	0.939	0.585	0.912	0.836	0.956	0.947	0.950	0.937	0.947	0.905	1.000	0.954	0.943	0.897	0.965
S12	0.931	0.625	0.922	0.873	0.955	0.947	0.947	0.920	0.972	0.884	0.954	1.000	0.956	0.934	0.983
S13	0.866	0.710	0.965	0.936	0.915	0.904	0.889	0.842	0.982	0.783	0.943	0.956	1.000	0.972	0.964
S14	0.851	0.673	0.949	0.953	0.908	0.898	0.875	0.808	0.965	0.733	0.897	0.934	0.972	1.000	0.955
*R*	0.943	0.621	0.950	0.903	0.974	0.964	0.957	0.913	0.985	0.867	0.965	0.983	0.964	0.955	1.000

*R*: compare to reference standard.

**Table 3:** The secretion and mRNA expression of IL-6 and IL-1*β* in microglial cells.

Group	ELISA		mRNA	
	IL-6	IL-1*β*	IL-6	IL-1*β*
Control	1.000	1.000	1.000	1.000
Model	1.421 ± 0.107^##^	1.788 ± 0.135^##^	3.054 ± 0.235^###^	3.609 ± 0.325^###^
S1	1.081 ± 0.067^∗^	1.230 ± 0.132^∗^	2.133 ± 0.181^∗^	2.590 ± 0.110^∗^
S2	1.016 ± 0.067^∗^	1.296 ± 0.155^∗^	2.667 ± 0.208	3.006 ± 0.250
S3	0.949 ± 0.039^∗∗^	1.178 ± 0.159^∗^	2.442 ± 0.161^∗^	2.459 ± 0.088^∗∗^
S4	1.052 ± 0.088^∗^	1.231 ± 0.075^∗∗^	1.793 ± 0.246^∗∗^	2.487 ± 0.201^∗^
S5	1.177 ± 0.083	1.233 ± 0.117^∗^	2.632 ± 0.169	2.324 ± 0.179^∗∗^
S6	1.050 ± 0.021^∗∗^	1.053 ± 0.083^∗∗^	2.307 ± 0.254^∗^	1.954 ± 0.281^∗∗^
S7	1.015 ± 0.058^∗∗^	0.978 ± 0.030^∗∗^	2.462 ± 0.134^∗^	2.188 ± 0.292^∗^
S8	1.204 ± 0.125	1.196 ± 0.149^∗^	2.539 ± 0.242	2.501 ± 0.139^∗^
S9	1.233 ± 0.127	1.162 ± 0.095^∗∗^	2.673 ± 0.241	2.340 ± 0.066^∗∗^
S10	1.014 ± 0.090^∗^	1.360 ± 0.063^∗^	1.868 ± 0.136^∗∗^	2.369 ± 0.133^∗∗^
S11	0.984 ± 0.023^∗∗^	1.192 ± 0.103^∗∗^	2.160 ± 0.156^∗^	2.247 ± 0.090^∗∗^
S12	0.968 ± 0.033^∗∗^	1.170 ± 0.016^∗∗^	2.036 ± 0.098^∗∗^	2.556 ± 0.320^∗^
S13	1.007 ± 0.117^∗^	1.184 ± 0.169^∗^	1.909 ± 0.172^∗∗^	2.281 ± 0.244^∗∗^
S14	0.913 ± 0.032^∗∗^	1.346 ± 0.112^∗^	2.067 ± 0.142^∗∗^	2.256 ± 0.317^∗^

^##^ *p* < 0.01, ^###^*p* < 0.001 were significant difference between the model group and control group; ^∗^*p* < 0.05, ^∗∗^*p* < 0.01 were considered statistically significant between the treatment groups and model group ($\bar{x}$ ± *s*, *n* = 3).

**Table 4:** The results of GRA between 20 characteristic peaks and inhibiting microglia activation.

Peak	ELISA		mRNA		Average	Rank
	IL-6 correlation	IL-1*β* correlation	IL-6 correlation	IL-1*β* correlation		
P1	0.967	0.968	0.966	0.969	0.967	2
P2	0.684	0.686	0.685	0.685	0.685	20
P3	0.968	0.967	0.966	0.968	0.967	4
P4	0.966	0.965	0.966	0.966	0.966	9
P5	0.968	0.967	0.966	0.969	0.967	3
P6	0.966	0.965	0.966	0.966	0.965	10
P7	0.958	0.958	0.958	0.958	0.958	15
P8	0.948	0.949	0.948	0.950	0.949	17
P9	0.967	0.966	0.966	0.967	0.966	6
P10	0.961	0.961	0.961	0.961	0.961	12
P11	0.966	0.966	0.965	0.967	0.966	8
P12	0.968	0.968	0.966	0.969	0.967	1
P13	0.955	0.955	0.954	0.956	0.955	16
P14	0.919	0.919	0.920	0.919	0.919	18
P15	0.853	0.853	0.853	0.853	0.853	19
P16	0.965	0.965	0.964	0.966	0.965	11
P17	0.960	0.959	0.960	0.959	0.959	14
P18	0.966	0.966	0.965	0.967	0.966	7
P19	0.966	0.967	0.965	0.968	0.966	5
P20	0.960	0.961	0.960	0.963	0.961	13

## Data Availability

All the data in this study are included within the article.

## Supplementary Materials

Supplementary Materials Table S1: sequence of IL-6 and IL-1*β* primers.
