# Supplementary material for: Spectrum-Efficacy Relationships between GC-MS Fingerprints of Essential Oil from Valerianae Jatamansi Rhizoma et Radix and the Efficacy of Inhibiting Microglial Activation
Source: Evid Based Complement Alternat Med. 2022 Mar 7;2022:9972902. doi: 10.1155/2022/9972902 (PMC8920623; doi:10.1155/2022/9972902)
Supplement: Supplementary Materials — Table S1: sequence of IL-6 and IL-1β primers. [file 9972902.f1.docx]

Table S1 Sequence of IL-6 and IL-1β primers

| Primers | Sequence（5’- 3’) |
| --- | --- |
| IL-6 | Forward sequence: ACTTCACAAGTCCGGAGAGG  Reverse sequence: TGCAAGTGCATCATCGTTGT |
| IL-1β | Forward sequence: GAAATGCCACCTTTTGACAGTG Reverse sequence: TGGATGCTCTCATCAGGACAG |
| GAPDH | Forward sequence: GGGTCCCAGCTTAGGTTCAT Reverse sequence: CCAATACGGCCAAATCCGTT |
